# High liver fibrosis scores in metabolic dysfunction-associated fatty liver disease patients are associated with adverse atrial remodeling and atrial fibrillation recurrence following catheter ablation

**DOI:** 10.3389/fendo.2022.957245

**Published:** 2022-08-31

**Authors:** Raphaël Decoin, Laura Butruille, Thomas Defrancq, Jordan Robert, Nicolas Destrait, Augustin Coisne, Samy Aghezzaf, Eloise Woitrain, Zouriatou Gouda, Sofia Schino, Cédric Klein, Patrice Maboudou, François Brigadeau, Didier Klug, Andre Vincentelli, David Dombrowicz, Bart Staels, David Montaigne, Sandro Ninni

**Affiliations:** ^1^ Univ. Lille, Inserm, CHU Lille, Institut Pasteur de Lille, U1011 - EGID, Lille, France; ^2^ CHU Lille, Institut Coeur-Poumon, Lille, France; ^3^ CHU Lille, Biochemistry Emergency, Lille, France; ^4^ CHU Lille, Service de Biochimie Automatisée Protéines, Lille, France

**Keywords:** MAFLD (metabolic associated fatty liver disease), atrial fibrillation, atrial remodeling, catheter ablation, liver fibrosis

## Abstract

**Background:**

A number of epidemiological studies have suggested an association between metabolic dysfunction-associated fatty liver disease (MAFLD) and the incidence of atrial fibrillation (AF). However, the pathogenesis leading to AF in the context of MAFLD remains unclear. We therefore aimed at assessing the impact of MAFLD and liver fibrosis status on left atrium (LA) structure and function.

**Methods:**

Patients with a Fatty Liver Index (FLI) >60 and the presence of metabolic comorbidities were classified as MAFLD+. In MAFLD+ patients, liver fibrosis severity was defined using the non-alcoholic fatty liver disease (NAFLD) Fibrosis Score (NFS), as follows: MAFLD w/o fibrosis (NFS ≦ −1.455), MAFLD w/indeterminate fibrosis (−1.455 < NFS < 0.675), and MAFLD w/fibrosis (NFS ≧ 0.675). In the first cohort of patients undergoing AF ablation, the structural and functional impact on LA of MAFLD was assessed by LA strain analysis and endocardial voltage mapping. Histopathological assessment of atrial fibrosis was performed in the second cohort of patients undergoing cardiac surgery. Finally, the impact of MAFLD on AF recurrence following catheter ablation was assessed.

**Results:**

In the AF ablation cohort (NoMAFLD n = 123; MAFLD w/o fibrosis n = 37; MAFLD indeterm. fibrosis n = 75; MAFLD w/severe fibrosis n = 10), MAFLD patients with high risk of F3–F4 liver fibrosis presented more LA low-voltage areas as compared to patients without MAFLD (16.5 [10.25; 28] vs 5.0 [1; 11] low-voltage areas p = 0.0115), impaired LA reservoir function assessed by peak left atrial longitudinal strain (19.7% ± 8% vs 8.9% ± 0.89% p = 0.0268), and increased LA volume (52.9 ± 11.7 vs 43.5 ± 18.0 ml/m^2^ p = 0.0168). Accordingly, among the MAFLD patients, those with a high risk of F3–F4 liver fibrosis presented a higher rate of AF recurrence during follow-up (p = 0.0179). In the cardiac surgery cohort (NoMAFLD n = 12; MAFLD w/o fibrosis n = 5; MAFLD w/fibrosis n = 3), an increase in histopathological atrial fibrosis was observed in MAFLD patients with a high risk of F3–F4 liver fibrosis (p = 0.0206 vs NoMAFLD; p = 0.0595 vs MAFLD w/o fibrosis).

**Conclusion:**

In conclusion, we found that liver fibrosis scoring in MAFLD patients is associated with adverse atrial remodeling and AF recurrences following catheter ablation. The impact of the management of MAFLD on LA remodeling and AF ablation outcomes should be assessed in dedicated studies.

## 1 Introduction

Non-alcoholic fatty liver disease (NAFLD) refers to a spectrum of liver diseases characterized by excessive hepatic fat accumulation, associated or not with liver inflammation and fibrosis with the exclusion of chronic alcohol consumption. Recently, a new concept of metabolic dysfunction-associated fatty liver disease (MAFLD) has been proposed to broaden the diagnostic criteria to meet the needs of a population not previously included in clinical studies (1). As such, the MAFLD definition includes alcohol consumption in the presence of at least one metabolic syndrome criterion (2, 3). Fatty liver diseases are the leading cause of chronic liver diseases in many western countries, with a worldwide estimated prevalence of approximately 25% (4). Several studies reported an epidemiological association between MAFLD and cardiovascular diseases, and pathophysiological mechanisms linking these two clinical entities have been recently suggested (5–7).

Although the liver–heart interaction is well described in the context of atherosclerosis (8) and ventricular remodeling (9), only sparse data explored the association between atrial pathology and MAFLD (9–11). A limited number of epidemiological studies have shown an independent association between MAFLD and the incidence of atrial fibrillation (AF) (12–14); however, the pathogenesis leading to AF in the context of MAFLD remains unclear.

AF pathogenesis encompasses a wide spectrum of mechanisms involving electrophysiological and structural remodeling of the left atrium (LA) (15). Furthermore, metabolic disorders have been previously associated with LA remodeling [e.g., mitochondrial dysfunction in diabetic patients (16–18) and enhanced LA fibrosis in the high-fat-diet-fed mice (19)] but did not emphasize the liver phenotype. Therefore, data assessing the impact of MAFLD on LA remodeling are severely lacking. Moreover, such insights might critically impact the management of patients presenting both AF and MAFLD, especially when invasive management of AF (i.e., using catheter ablation) is considered.

We hypothesized that MAFLD is associated with poor LA remodeling and might thus alter outcomes associated with AF catheter ablation. To explore this hypothesis, we first assessed the impact of MAFLD on LA structure and function (as assessed by LA echocardiographic parameters, endocardial electrophysiological mapping, and histopathological assessment of LA fibrosis) using two distinct cohorts of patients. Then, the impact of MAFLD on AF recurrence following catheter ablation was assessed.

## 2 Materials and methods

### 2.1 Study populations

#### 2.1.1 Catheter ablation cohort

Between March 2018 and April 2021, all patients who were candidates for a first AF catheter ablation in the Lille University Hospital were retrospectively enrolled. As specified in the 2016 European Society of Cardiology (ESC) Guidelines for the management of AF, patients were eligible for catheter ablation if they had new-onset or under-treated paroxysmal or persistent AF (20). Patients with prior AF catheter ablation or severe valvular heart disease were excluded. The protocol was approved by the local ethics committee, and patients gave informed consent.

#### 2.1.2 POMI-AF (NCT03376165)

The cohort study population consisted of consecutive patients (aged ≥18 years) undergoing cardiac surgery referred to the Cardiovascular Surgery Department at the Lille University Hospital (Lille, France) for aortic valve replacement or mitral valve repair (with or without coronary artery bypass graft). Patients with another notable valvular disease, a medical history of previous cardiac surgery, or congenital heart diseases were excluded. The ethics committee of our institution approved the cohort protocol. Written informed consent was obtained from all patients before inclusion in this cohort.

### 2.2 Diagnosis of metabolic dysfunction-associated fatty liver disease and severity assessment

As recommended by the current guidelines (21) and taking into account other metabolic disorders listed below (including alcohol consumption) (1), MAFLD was diagnosed using the Fatty Liver Index (FLI), which was calculated using blood triglyceride levels, body mass index (calculated as the weight in kilograms divided by the height in square meters), gamma-glutamyl transpeptidase (GGT) activity in blood, and waist circumference (in centimeters) according to (22, 23) the following: 0.953 × ln(triglycerides, mg/dl) + 0.139 × BMI, kg/m^2^ + 0.718 × ln(GGT, U/L) + 0.053 × waist circumference, cm − 15.745. Patients with an FLI score of 60 or higher with metabolic syndrome (waist circumference >94 cm in men or >80 in women associated with at least two of the following parameters: triglyceridemia > 1.5 g/L; high-density lipoprotein cholesterol (HDL-c) <0.4 g/L in men or <0.5 g/L in women; fasting glycemia > 1 g/L; systolic arterial blood pressure > 130 mmHg and/or diastolic blood pressure > 85 mmHg) (24) were considered as having MAFLD. To assess the severity of hepatic fibrosis, the validated NAFLD fibrosis score (NFS) in patients with positive diagnoses of MAFLD (3) was used. The NFS was assessed as follows: [−1.675 + 0.037 × age (years) + 0.094 × body mass index (kg/m^2^) + 1.13 × diabetes mellitus (if presence, given 1) + 0.95 × aspartate transaminase (AST) (U/L) to alanine aminotransferase (ALT) (U/L) ratio − 0.013 × platelet count (10^−9^/L) − 0.66 × albumin (mg/L)]. According to the original validation work, patients with an NFS of 0.675 or higher were considered positive for advanced liver fibrosis. Patients presenting a NAFLD fibrosis score below −1.455 were considered free from advanced liver fibrosis. A score between 0.675 and −1.455 was considered indeterminate (25, 26). For each patient, insulin resistance was estimated by Homeostatic Model Assessment for Insulin Resistance (HOMA-IR) calculation ([fasting insulin (μU/ml) × fasting glucose (mmol/L)]/22.5).

### 2.3 Atrial fibrillation ablation procedure and low-voltage area exploration

All procedures were performed under local anesthesia and conscious sedation using intravenous boluses of morphine. Left atrial reconstruction was performed using a CARTO3 (Biosense Webster^®^, Irvine, CA, USA) electroanatomic mapping system. Mapping of the pulmonary veins was performed with a deflectable decapolar catheter (Lasso, Biosense Webster^®^). Ablation was performed using a 3.5-mm irrigated ablation catheter with contact force sensing (ThermoCool, Biosense Webster^®^). A third, standard quadripolar catheter was used for reference and placed into the coronary sinus. For all procedures, and according to current guidelines, pulmonary vein isolation was performed in both paroxysmal and persistent AF and cavotricuspid isthmus ablation in case of typical atrial flutter. Additional lines (superior vena cava isolation, left atrial roof line, and other left atrial endocardial lines), voltage mapping, or recording of continuous fragmented atrial electrograms (CFAEs) to target ablation were deployed to consultant conviction. For patients with left atrial voltage mapping, data were collected at the beginning of the AF ablation procedure in sinus rhythm, using an electroanatomic mapping (EAM) system (CARTO3, Biosense Webster) and a mapping catheter with a 3.5-mm distal tip and a 2-mm inter-electrode spacing (NaviStar, Thermocool Smartouch, Biosense Webster, Inc.). Adequate endocardial contact was confirmed by stable electrograms and increased contact force values of ≥10 g. The left atrium was divided into nine regions, i.e., septum, superior, posterior, inferior, and lateral walls and the four pulmonary veins (PVs) at their antrum (**Supplementary S1**). In each predefined region, at least 12 voltage-mapping points were collected. A low-voltage zone (LVZ) was defined as a region with bipolar voltage of less than 0.5 mV in patients in sinus rhythm at the time of point acquisition. The low-voltage area was calculated as the following ratio: point displaying voltage <0.5 mV/total point acquires.

### 2.4 Follow-up and recurrence assessment

All patients underwent a clinical follow-up (up to 3 years). First, AF recurrence was established as documented AF or atrial flutter on 12-lead electrocardiogram and/or episode >30 s during Holter monitoring. Within the first year after ablation, our in-house protocol unfolds electrocardiograms when symptoms are reported, during outpatient visits in our tertiary hospital, or in the treating cardiologist’s office (at 3, 6, and 12 months). Holter monitoring at 24 to 48 h was also performed at 3 and 6 months. The treating cardiologists then followed up the patients, with the number of outpatient visits at clinician discretion. A 90-day “blanking period” was followed. Follow-up of “MAFLD” vs “NoMAFLD” subgroups was censored at 1,200 days, and follow-up of “MAFLD” patients according to liver fibrosis risk was censored at 400 days because the sample size dropped to only one patient.

### 2.5 Histological analysis

Atrial biopsies were recovered during the cardiac operation in POMI-AF patients. Biopsies were then processed for paraffin embedding. Paraffin-embedded sections were stained with Sirius Red (RAL ref.363440-0005), and images were captured using a ZEISS Axio Scan.Z1 slide scanner. Collagen surface assessment was performed using ImageJ software (version 2.1.0/1.53C for Windows) on the entire image surface, except for the epicardium. The area corresponding to collagen was divided by the total area of the quantified surface to obtain the ratio of fibrosis.

### 2.6 Statistics

Continuous variables were tested for normality with the Shapiro test and were represented as mean ± SD in case of normality, or median [interquartile range (IQR)] otherwise. Continuous variables with no Gaussian distribution are given as median (IQR). Categorical variables were given as percentages of individuals. Bivariate comparisons were performed using the t-test for normally distributed continuous variables or the Mann–Whitney U test for variables not normally distributed. Bivariate comparisons of categorical variables were performed with the χ^2^ test. When expected counts were lower than 5, Fisher’s exact test was used. Event-free survival was estimated by the Kaplan–Meier method. First, the association between baseline characteristics and outcomes was investigated in MAFLD and non-MAFLD patients separately. The association between liver fibrosis and AF recurrence was then investigated according to the three groups defined by the MAFLD fibrosis score (no or mild fibrosis, indeterminate score, and severe fibrosis or cirrhosis). The baseline characteristics between the three groups were compared by one-way ANOVA for normally distributed variables or the Kruskal–Wallis test for not normally distributed variables. Second, the association between liver fibrosis and AF recurrence was investigated after adjustment confounders [namely, subtype of AF, age, left atrial size, BMI, and sex] in separate multivariate proportional hazards Cox models. A two-tailed p-value <0.05 was considered statistically significant. All analyses were performed using MedCalc v16.4 (Ostend, Belgium). Visual rendering of the graphics was performed using GraphPad Prism version 9.0.0 for Windows (GraphPad Software, San Diego, CA, USA).

## 3 Results

### 3.1 Metabolic dysfunction-associated fatty liver disease classifications and baseline characteristics

A total of 291 patients referred to our center for a first AF ablation were included from March 2017 to December 2021. After exclusion of patients with missing components of non-invasive hepatic fatty liver or fibrosis scores, and/or no available follow-up for AF recurrence, 245 were available for analysis. With the use of the FLI cutoff of 60 (27), 123 patients were classified as free from MAFLD (“NoMAFLD” group) and 122 as having MAFLD (MAFLD group). Comparing “NoMAFLD” to “MAFLD” (**Table 1**), no differences were observed regarding age (60 ± 10 vs 58 ± 10 years). However, cardiovascular comorbidities were more prevalent in the MAFLD group, such as hypertension (29% vs 55% p = 0.0001) or heart failure (20% vs 30% p = 0.083). Half of the procedures were performed for paroxysmal AF. Interestingly, MAFLD patients displayed LA dilatation with an increased area (23 [19; 26] cm^2^ vs 28 [24; 31] cm^2^, p < 0.0001).

**Table 1:** Baseline characteristics According to MAFLD status.

	noMAFLD (n = 123)	MAFLD (n=122)	P
*Criteria*	FLI<60	FLI≧60	
*Age (years)*	60.3±10.2	58.1±9.9	0.089
*Women, n (%)*	56 (46%)	29 (23%)	**0.0004**
*BMI (kg/m²)*	24.7±3.0	31.4±4.5	<**0.0001**
*Chronic alcohol consumption, n (%)*	9 (7%)	22 (18%)	**0.023**
*Waist circumference (cm)*	91.3±9.6	112.0±12.1	**<0.0001**
*Hypertension, n (%)*	36 (29%)	68 (55%)	**0.0001**
*Diabetes mellitus, n (%)*	15 (12%)	27 (22%)	0.067
*CHa_2_DS_2_Vasc*	1 [1;3]	2 [1;3]	0.409
*Paroxysmal AF, n (%)*	72 (59%)	51 (41%)	**0.004**
*Persistent AF, n (%)*	51 (41%)	69 (56%)	
*PVI alone, n (%)*	100 (83%)	92 (79%)	0.536
*PVI + lines, n (%)*	21 (17%)	25 (21%)	
*AAD at discharge*	22 (17.9%)	34 (27.8%)	0.0789
*Flecainide*	12 (55%)	13 (38%)	
*Sotalol*	4 (18%)	4 (12%)	
*Amiodarone*	6 (27%)	17 50%)	
*History of heart failure, n (%)*	24 (20%)	37 (30%)	0.083
*LVEF – normal range, n (%)*	58 (48%)	38 (31%)	**0.0075**
*LVEF – mildly abnormal range, n (%)*	60 (50%)	76 (61%)	
*LVEF – abnormal range, n (%)*	3 (2%)	10 (8%)	
*Left atrium area (cm²)*	23 [19;26]	28 [24;31]	**<0.0001**
*Fasting glucose (mg/dl)*	98±15	108±18	**<0.0001**
*HOMA IR*	1.34 [0.89;2.06]	2.45 [1.65;3.45]	**<0.0001**
*Triglycerides (g/l)*	90 [72;116]	137 [106;182]	**<0.0001**
*ASAT (UI/l)*	23 [18;26]	24 [18;30]	**0.0274**
*ALAT (UI/l)*	20 [16;26]	27 [21;37]	**<0.0001**
*Gamma GT (UI/l)*	24 [18;34]	47 [32;86]	**<0.0001**
*Albumin (g/l)*	38 [36;42]	39 [36;42]	0.906
*Nt pro BNP (ng/l)*	209 [97;487]	294 [115;659]	0.198

Statistics, for continuous variables with normal distribution, Student’s t-test; for continuous variables without normal distribution, Mann-Whitney U test; for frequencies, chi-squared test; for frequencies with linear trends (AF subtype; PVI ± other lines; LVEF) chi-squared for trend. MAFLD, Metabolic-dysfunction Associated Fatty Liver Disease; BMI, Body Mass Index; AF, Atrial Fibrillation; PVI, Pulmonary Vein(s) Isolation; AAD, Anti-Arrhythmic Drug; LVEF, Left Ventricular Ejection Fraction; FLI, Fatty Liver Index. Norms, chronic alcohol consumption >40 g per day in men, and >20 g per day in women 28; LVEF normal range ≥ 50%; mildly normal range 41%–49%; normal range ≤ 40% 29. P-values lower than 0.05 are represented in bold.

In the MAFLD group, the NFS was used to dichotomize patients at risk for severe liver fibrosis (n = 10; “MAFLD w/fibrosis”), patients with an undetermined risk for liver fibrosis (n = 75; “MAFLD indeterm. fibrosis”), and patients not at risk for liver fibrosis (n = 37; “MAFLD w/o fibrosis”). The full flowchart is provided in **Figure S2**. Baseline characteristics of the three resulting groups are shown in **Table 2**. Patients in the “MAFLD w/fibrosis” group were older (52 ± 11 for “MAFLD w/o fibrosis” vs 60 ± 8 for “MAFLD indeterm. fibrosis” vs 67 ± 4 years for “MAFLD w/fibrosis” p < 0.001) and had higher HOMA-IR (1.99 [1.45; 2.60] for “MAFLD w/o fibrosis” vs 2.65 [1.89; 3.58] for “MAFLD indeterm. fibrosis” vs 3.44 [1.65; 4.43] for “MAFLD w/fibrosis” p = 0.029). Thus, the NFS identified a subgroup of patients exhibiting a poor metabolic profile.

**Table 2:** Baseline characteristics of adults with MAFLD diagnosis classified according to liver fibrosis risk using the NAFLD Fibrosis Score (NFS).

	*No or mild fibrosis (n = 37)*	*Indeterminate(n = 75)*	*Severe fibrosis (n = 10)*	p
Criteria	FLI ≧ 60 NFS < −1.455	FLI ≧ 60 −1.455 < NFS < 0.675	FLI ≧ 60 NFS ≧ 0.675	
Age (years)	52 ± 11	60 ± 8	67 ± 4	**<0.001 (#Ø)**
Women, n (%)	10 (26%)	15 (19%)	4 (40%)	0.319
BMI (kg/m^2^)	30.0 ± 3.6	32.1 ± 4.6	31.5 ± 6.1	0.056
Chronic alcohol consumption, n (%)	4 (10.5%)	14 (18.4%)	4 (40%)	0.092
Waist circumference (cm)	108 ± 11	114 ± 12	108 ± 11	**0.042 (#)**
Hypertension, n (%)	15 (40%)	44 (58%)	9 (90%)	**0.012 (Ø)**
Diabetes mellitus, n (%)	3 (8%)	19 (25%)	5 (50%)	**0.009 (Ø)**
CHa_2_DS_2_Vasc	1 [0; 2]	2 [1; 3]	4 [3; 4]	**<0.001 (Ø)**
Paroxysmal AF, n (%)	23 (60%)	25 (33%)	3 (30%)	0.115
Persistent AF, n (%)	14 (37%)	48 (63%)	7 (70%)	
PVI alone, n (%)	26 (74%)	60 (83%)	6 (60%)	0.182
PVI + lines, n (%)	9 (26%)	12 (17%)	4 (40%)	
AAD at discharge	11 (29.7%)	21 (27.6%)	2 (20%)	0.5919
Flecainide	7 (64%)	6 (29%)	0 (0%)	
Sotalol	1 (9%)	3 (14%)	0 (0%)	
Amiodarone	3 (27%)	12 (57%)	2 (100%)	
History of heart failure, n (%)	7 (18%)	29 (38%)	1 (10%)	**0.034 (#)**
LVEF—normal range, n (%)	32 (84%)	51 (67%)	8 (80%)	0.078
LVEF—mildly normal range, n (%)	6 (16%)	11 (15%)	1 (10%)	
LVEF—normal range, n (%)	0 (0%)	14 (18%)	1 (10%)	
Fasting glucose (mg/dl)	101 ± 8	110 ± 20	119 ± 27	**0.005 (#Ø)**
HOMA IR	1.99 [1.45; 2.60]	2.65 [1.89; 3.58]	3.44 [1.65; 4.43]	**0.029 (#Ø)**
Triglycerides (g/L)	135 [111; 167]	144 [107; 197]	109 [101; 177]	0.221
ASAT (UI/L)	25 [19; 30]	24 [18; 30]	27 [18; 34]	0.662
ALAT (UI/L)	30 [23; 43]	27 [19; 35]	24 [15; 33]	0.052
Gamma GT (UI/L)	49 [33; 80]	45 [30; 85]	63 [42; 153]	0.220
Albumin (g/L)	40 [37; 42]	39 [36; 41]	35 [32; 39]	**0.023 (Ø)**
NT-pro-BNP (ng/L)	205 [58; 595]	313 [138; 659]	472 [232; 1,257]	0.164

Statistics, for continuous variables with normal distribution, Student’s t-test; for continuous variables without normal distribution, Mann–Whitney U test; for frequencies, chi-squared test; for frequencies with linear trends (AF subtype; PVI ± other lines; LVEF) chi-squared for trend.

MAFLD, metabolic dysfunction-associated fatty liver disease; BMI, body mass index; AF, atrial fibrillation; PVI, pulmonary vein isolation; AAD, anti-arrhythmic drug; LVEF, left ventricular ejection fraction; FLI, fatty liver index.

Norms: chronic alcohol consumption >40 g per day in men and >20 g per day in women 28; LVEF normal range ≧ 50%; mildly normal range 41%–49%; normal range ≦ 40% 29.

Symbols: #, significant no or mild fibrosis vs indeterminate; Ø, significant no or mild fibrosis vs severe fibrosis. P-values lower than 0.05 are represented in bold.

### 3.2 Left atrial structural and functional remodeling according to non-alcoholic fatty liver disease fibrosis score

**Figure 1:**
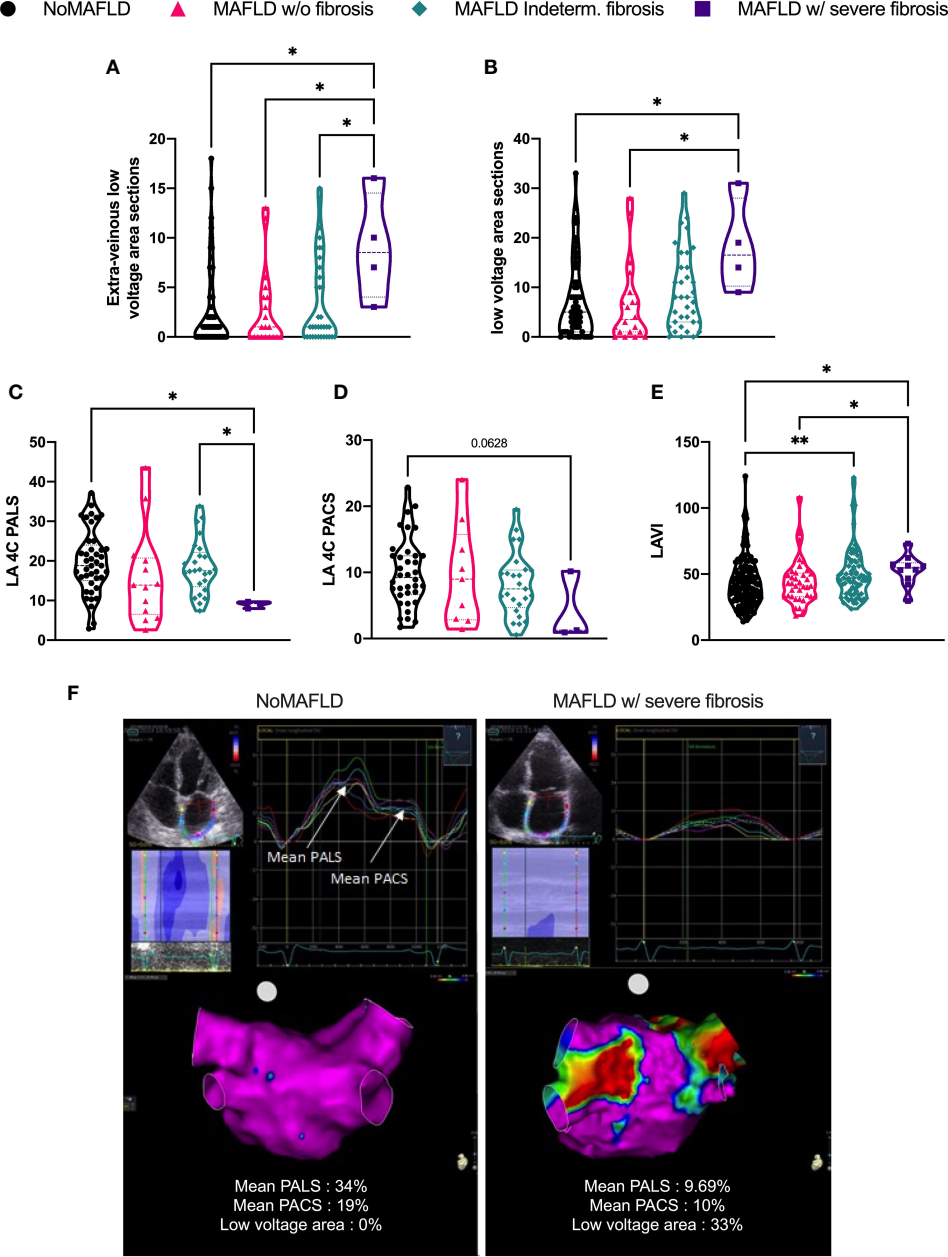
Left atrial structural and electrical remodeling parameters. Number of low-voltage area extra-venous **(A)** or total **(B)**; peak atrial longitudinal strain **(C)** and peak atrial contraction strain **(D)**; left atrial volume indexed to body surface area **(E)**. Representative echography, strain values, and bipolar voltage maps (low-voltage cutoff: 0.5 mV) **(F)**. NoMAFLD: FLI < 60; MAFLD w/o fibrosis: FLI > 60 and NFS < 1.455; MAFLD ind. fibrosis: FLI > 60 and −1.455 < NFS < 0.675; MAFLD w/severe fibrosis: FLI > 60 and NFS > 0.675. Kruskal–Wallis test followed by Dunn’s post-hoc test. *p < 0.05; **p < 0.01. MAFLD, metabolic dysfunction-associated fatty liver disease; LA, left atria; PALS, peak atrial longitudinal strain; PACS, peak atrial contraction strain; LAVI, left atrial volume index.

Structural and function atrial remodeling were explored in this cohort using 3D-voltage mapping and LA echographic parameters. A total of 183 patients were explored in sinus rhythm with 3D-voltage mapping. Of note, the median left atrial low-voltage area was 0% [0; 10]. Therefore, most patients presented no or a limited low-voltage area. However, the “MAFLD w/severe fibrosis” patients presented an increase in extra-venous low-voltage area sections (**Figure 1A**), in total low-voltage area sections (**Figure 1B**) compared to the three other groups (e.g., total low-voltage area sections: “NoMAFLD” = 5 [1; 11] vs “MAFLD w/severe fibrosis” = 16.5 [10.25; 28] p = 0.0115). The two remaining MAFLD subgroups (“MAFLD indeterm. fibrosis” and “MAFLD w/o fibrosis”) did not display any significant difference regarding low-voltage area compared to “noMAFLD” patients. Since low-voltage zones have been associated with local fibrosis (30), which may impact LA hemodynamics, we explored the LA reservoir and contractile function assessed by four-chamber LA 2D-speckle tracking in patients presenting sinus rhythm prior to AF ablation.

The LA reservoir function, as assessed by the mean peak left atrial longitudinal strain (PALS), was significantly reduced in the “MAFLD w/severe fibrosis” group compared to the “NoMAFLD” group (**Figure 1C**, 19.7% ± 8% vs 8.9% ± 0.89% p = 0.0268). Furthermore, a trend toward a decrease in the peak atrial contraction strain (PACS) was also observed in the “MAFLD w/severe fibrosis” compared to the “NoMAFLD” group (**Figure 1D**, 10.0% ± 5.1% vs 4.1% ± 5.2% p = 0.0628). Taken together, these data indicated an altered LA compliance and a trend toward a depressed contractibility of the LA in patients presenting “MAFLD w/severe fibrosis”. Accordingly, an increased LA volume was observed with the LA volume index (LAVI) (**Figure 1E**, 43.5 ± 18 ml/m^2^ in “NoMAFLD” vs 52.9 ml/m^2^ in “MAFLD w/severe fibrosis”, p = 0.0168) and 50.4% of the “NoMAFLD” group vs 90% in the “MAFLD w/severe fibrosis group” presenting severe LA dilatation (p = 0.0196). Representative examples of atrial function and voltage according to MAFLD fibrosis status are provided in **Figure 1F**. Taken together, these data suggest that increased NFS in a MAFLD population detected more pronounced LA remodeling in patients, which resulted in altered electrophysiological and hemodynamic properties.

### 3.3 Histological assessment of atrial remodeling

**Figure 2:**
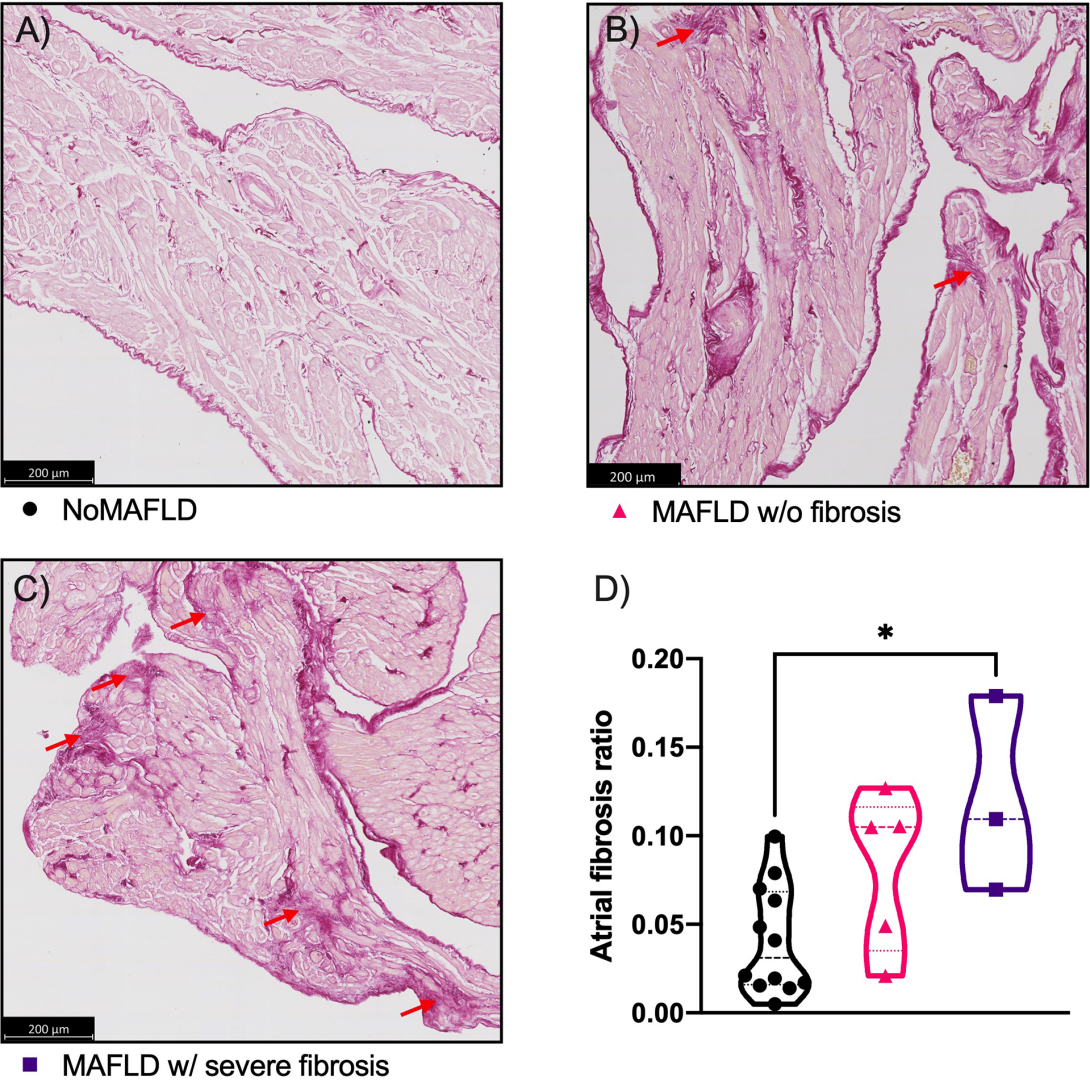
Histological analysis of atrial fibrosis in patient biopsies. Representative images of atrial fibrosis in NoMAFLD patient **(A)**; MAFLD w/o fibrosis **(B)**; MAFLD w/severe fibrosis **(C)**. Red arrows indicate fibrotic foci. Semi-automatic quantification: ratio between positive fibrotic area and total selected area **(D)**. NoMAFLD: FLI < 60; MAFLD w/o fibrosis: FLI > 60 and NFS < 1.455; MAFLD w/severe fibrosis: FLI > 60 and NFS > 0.675. Kruskal–Wallis test followed by Dunn’s post-hoc test. *p < 0.05. MAFLD, metabolic dysfunction-associated fatty liver disease.

The above data suggest an increase in atrial fibrosis in patients presenting MAFLD with severe liver fibrosis. We therefore explored the presence of atrial fibrosis according to MAFLD status in atrial biopsies collected in the second cohort of patients undergoing cardiac surgery (POMI-AF NCT03376165). In this cohort, a total of 20 patients underwent right atrial appendage biopsies during cardiac surgery and were classified according to the same FLI and NFS cutoff levels. Their baseline characteristics are summarized in **Table S1**. The histological analysis (using Sirius-Red coloration) of the 20 biopsies revealed atrial fibrosis patches (**Figure 2**). Of note, the fibrosis patches originated from capillary vessels, as frequently observed in the fibrotic process. After a semi-automatic quantification, patients in the “MAFLD w/severe fibrosis” had an average 2.78-fold increase in atrial fibrosis as compared to “NoMAFLD” patients (p = 0.0206). Accordingly, these patients tend to display higher LA areas (**Table S1**, 24 [22.5–29.5] vs 41 [24–45] cm^2^, p = 0.0847). Thus, in line with the AF ablation cohort, an increased NFS among MAFLD patients is associated with more severe structural atrial remodeling.

### 3.4 Atrial fibrillation ablation recurrence according to metabolic dysfunction-associated fatty liver disease and liver fibrosis status

Since atrial remodeling might critically impact the outcomes of AF ablation, the impact of MAFLD status on AF recurrence following ablation in the AF ablation cohort was assessed. In 201 out of 245 patients, lone pulmonary vein isolation (PVI) was performed. For the remaining 46 patients, additional lesions were assessed and included roof lines for 19 patients, cavotricuspid isthmus lines for 19 patients, box isolation for six patients, and other lines for 15 patients. Of note, at the end of the procedure, PVI was achieved in 100% of the patients.

**Figure 3:**
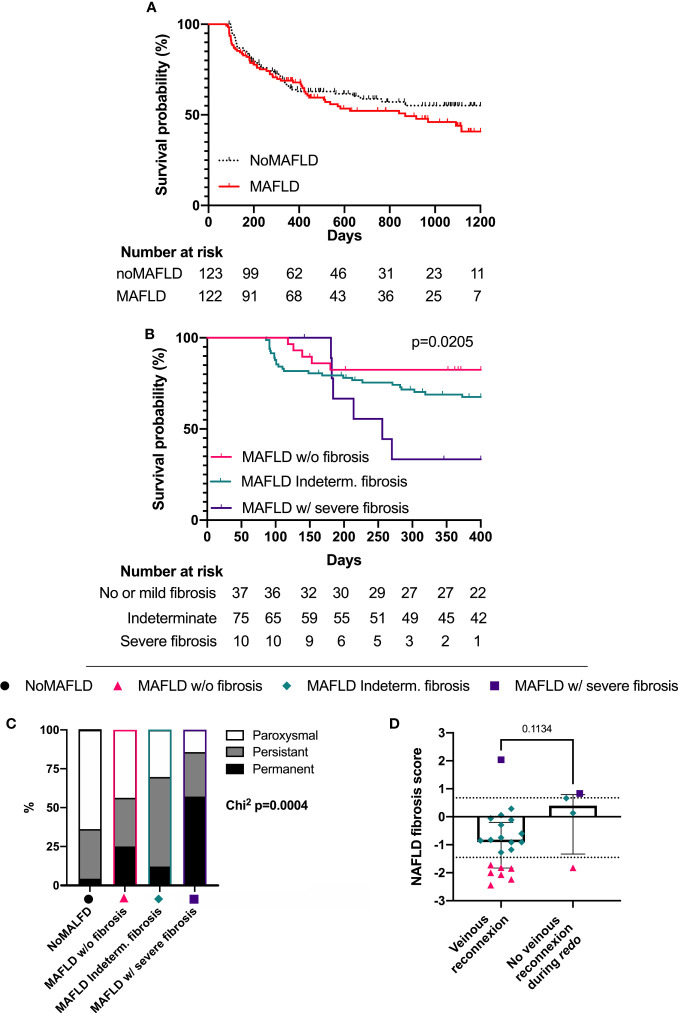
AF recurrence burden according to liver status. Atrial fibrillation recurrence after first ablation according to the MAFLD status **(A)** and liver fibrosis status in MAFLD patients **(B)**. Atrial fibrillation recurrence subtype (NoMAFLD n = 47; MAFLD w/o fibrosis n = 16; MAFLD ind. fibrosis n = 33; MAFLD w/severe fibrosis n = 7) **(C)** and recurrence mechanism according to the NAFLD Fibrosis Score **(D)**. NoMAFLD: FLI < 60; MAFLD w/o fibrosis: FLI > 60 and NFS < 1.455; MAFLD ind. fibrosis: FLI > 60 and −1.455 < NFS < 0.675; MAFLD w/severe fibrosis: FLI > 60 and NFS > 0.675. Survival analysis: log-rank (Mantel–Cox) if two groups **(A)** or log-rank for trend if more than two groups **(B)**; chi-squared for trend **(C)**; Mann–Whitney test **(D)**. Median ± interquartile range ; dashed lines represent the validated NFS cutoffs of −1.455 and 0.675 **(D)**. MAFLD, metabolic dysfunction-associated fatty liver disease; NAFLD, non-alcoholic fatty liver disease.

After a median follow-up of 418 days [197; 868], AF recurrence occurred in 42.8% of the patients. No significant difference in AF recurrence was observed according to MAFLD status alone (54% in MAFLD patients vs 41% in noMAFLD patients at 1,200 days; log-rank: p = 0.3093; **Figure 3A**). The impact of liver fibrosis on AF recurrence was then assessed in the three MAFLD groups (“MAFLD w/o fibrosis”, “MAFLD indeterm. fibrosis”, and “MAFLD w/severe fibrosis”). AF-free survival curves, following a first intervention, up to 400 days are shown in **Figure 3B**. During follow-up, the “MAFLD w/severe fibrosis” patients presented a higher rate of AF recurrence (77%) than patients with indeterm. fibrosis (32.5%) or without hepatic fibrosis (17.5%) according to NFS (log-rank for trend p = 0.0179; “MAFLD w/o fibrosis” vs “MAFLD w/severe fibrosis” hazard ratio (HR) = 5.345, 95% CI [1.335–21.40]) (**Figure 3B**). Furthermore, the non-adjusted Cox regression model (**Table 3A**) identified NFS as predictive of AF recurrence (p = 0.0184). After adjustment to the LA area and AF subtype (known non-covariable risk factors), the NFS remained significantly predictive of AF recurrence (**Table 3B**).

**Table 3:** Cox regression analysis of AF recurrence after ablation.

Models	Variables	p	b (SE)	HR [95% CI]
A) Unadjusted	NFS (continuous)	**0.0184**	1.35 (0.15)	–
B) Adjusted	NFS (continuous)	**0.023**	1.37 (0.16)	–
	LAA (cm^2^)	0.212	0.04 (0.03)	–
	Atrial fibrillation subtype	0.061	–	1.59 [0.98–2.58]
	Sex	0.657	–	0.83 [0.37–1.85]

NFS, NAFLD fibrosis score; LAA, left atrial area; NAFLD, non-alcoholic fatty liver disease. P-values lower than 0.05 are represented in bold.

In addition to the incidence of AF recurrence, the characteristics of AF recurrence according to MAFLD status were further explored. During the follow-up, 103 patients presented AF recurrences. Of note, 48 of this recurrence occurred as paroxysmal AF, 41 as persistent AF, and 14 as permanent AF. Moreover, the “MAFLD w/severe fibrosis” group presented more frequently AF recurrence as a permanent AF subtype. Conversely, recurrences as paroxysmal AF were gradually less frequent in the “MAFLD w/severe fibrosis” group (**Figure 3C**, chi-squared: p = 0.0004). Accordingly, MAFLD patients with severe liver fibrosis were less likely to undergo a second AF ablation (=*redo*) after recurrence, in comparison to MAFLD patients without severe liver fibrosis (chi-squared-for-trend: p = 0.051, **Figure S3A**).

To gain insight into the mechanisms leading to AF recurrence in “MAFLD w/severe fibrosis” patients, the electrophysiological findings observed during *redo* procedures were further explored. During follow-up, 25 *redo* procedures were performed. During *redo* procedures, venous reconnections were observed in 84% of patients. Interestingly, patients presenting AF recurrence without venous reconnection tended to have higher NFS as compared to patients presenting venous reconnection (**Figure 3D**).

Taken together, these data suggest that patients presenting MAFLD and severe liver fibrosis exhibit a higher risk for AF recurrence after ablation. Furthermore, these recurrences are more likely associated with a higher AF burden.

## 4 Discussion

Cardiac remodeling, AF, and metabolic disorders are closely intertwined. Furthermore, MAFLD has been recently suggested as a potential actor in the AF pathogenesis of patients exhibiting metabolic syndrome (10).

In this study, we showed that i) the presence of MAFLD is associated with adverse atrial remodeling as assessed by echocardiographic, electrophysiological, and histopathological analysis. More precisely, we showed a structural remodeling as indicated by the increase in LA volume, impaired LA reservoir function, and increased low-voltage areas in MAFLD patients at risk of liver fibrosis. Accordingly, atrial fibrosis was increased in MAFLD patients at risk of liver fibrosis. ii) The liver fibrosis scoring in MAFLD patients was predictive of AF recurrence after ablation. iii) In the case of AF recurrence, MAFLD patients with high liver fibrosis scores presented a higher AF burden.

### 4.1 Atrial remodeling

Atrial remodeling can be characterized by any complex structural, architectural, contractile, or electrophysiological changes affecting the atria with the potential to produce clinically relevant manifestations (31). Such hallmarks of the LA remodeling process were recently defined by the concept of atrial cardiomyopathy (15). Beyond its pathophysiological value, accumulating works highlight the clinical relevance of LA remodeling in order to manage AF and non-AF patients (32, 33).

It is now well recognized that metabolic disorders critically affect the course of patients presenting AF in terms of AF incidence (34), stroke risk (35), and rhythm management (36). Therefore, understanding LA remodeling in the context of metabolic disorders represents a major opportunity to improve metabolic patients’ care. However, the mechanisms leading to LA remodeling in the context of metabolic disorders remain incompletely understood.

Although metabolic disorders were first believed to directly induce LA remodeling and AF (37), recent data highlighted the potential role of MAFLD as a candidate involved in the pathogenesis of AF in patients presenting metabolic disorders. Such a mechanism was first suggested by epidemiological studies associating NAFLD and the incidence of AF. Targher et al. published two studies involving type 2 diabetes patients who had an independent association between NAFLD and AF development (adjusted OR: 5.88; 95% CI: 2.72 to 12.7 and 6.38; 95% CI: 1.7 to 24.2) (13, 38). Similarly, Käräjämäki et al. published a prospective cohort study of 958 hypertensive patients, which demonstrated an independent association between NAFLD and AF (adjusted OR: 1.88; 95% CI: 1.03 to 3.45) (39).

Despite these established epidemiological observations, mechanistic approaches are severely lacking. Recently, a study published by Suffee *et al.* using a high-fat-diet mouse model showed a vulnerability to AF linked to a shorter action potential duration caused by enhanced K-ATP current in the context of obesity (19). Although interesting, such studies did not emphasize the liver phenotype so far.

A precise assessment of LA remodeling in MAFLD patients was not performed so far to the best of our knowledge. Of note, assessing LA remodeling in patients requires a multidimensional approach to qualify the hemodynamic, histopathological, and electrophysiological parameters of the atria.

In the AF ablation cohort, the hemodynamic assessment of the LA was performed using echocardiographic speckle-tracking, which was previously correlated with LA hemodynamic profile (40). Importantly, we observed a reduced reservoir and contractile function in a patient with a high probability of liver fibrosis (“MAFLD w/fibrosis”). Such hemodynamic alteration might critically impact patients’ outcomes since impaired PALS was previously associated with increased risk for AF, stroke, and heart failure (41).

The electrophysiological remodeling was also observed with an increase in bipolar low-voltage areas in patients presenting MAFLD at high risk of liver fibrosis. Low-voltage areas act as arrhythmogenic substrates, promoting more persistent AF by slowing electrical conduction and sustaining fibrillatory conduction (42–44). Furthermore, low-voltage areas were identified as predictors of AF recurrence following AF ablation and associated with an increased risk for stroke (45). Therefore, the increased prevalence of low-voltage areas in MAFLD patients might severely impact outcomes following ablation.

In our study, the presence of atrial fibrosis in patients with MAFLD was specifically assessed using histopathological assessment of right atrial appendages biopsies. Interestingly, we found that atrial fibrosis was particularly increased in patients presenting MAFLD with high liver fibrosis scoring. Atrial fibrosis is a dominant factor for the development of AF (46) and is promoted by several clinical conditions such as heart failure or hypertension (47) and AF itself (48).

Taken together, our data suggest a fibrotic-mediated LA remodeling according to MAFLD and liver fibrosis classification. However, the mechanisms leading to such a fibrotic process need to be clarified. Atrial and liver fibrosis mechanisms share some similar pathogenic mechanisms, upstream to fibroblast activation within the myocardium or its counterpart, the hepatic stellate cell (HSC), in the liver. These cells, as activated myofibroblast, contribute to the majority of collagen formation (49, 50). In the liver, recruited or resident macrophages are known to trigger HSC activation through TGFβ (51). Similarly in the heart, macrophages are thought to be a major actor in fibroblast activation (52). Thus, if we consider MAFLD as a systemic low-grade inflammation, fibrosis development may be due to prior immune cell activation within the heart. Moreover, in our study, short-term MAFLD (i.e., with low liver fibrosis probability) is not associated with atrial fibrosis, making the early metabolic dysfunction hypothesis alone insufficient in our population.

The visceral adipose tissue was also suggested as a major actor in atrial remodeling. More particularly, epicardial adipose tissue volume has been associated with AF incidence and ablation outcome (53–56). The potential role of adipokines in myocardial remodeling was also highlighted by previous studies. In a cohort of 94 patients, the circulating adiponectin level was inversely correlated with indexed left ventricular mass (57). In line, a positive correlation was observed between indexed left atrial volume and E/e′ ratio with serum adiponectin levels (58). These clinical findings are also supported by translational studies suggesting the importance of adipokines on atrial myocardium (59, 60).

Patients presenting MAFLD with liver fibrosis display major alterations in adipokine profile such as lower adiponectin (61) levels or increased leptin levels (62), in comparison to patients without morbid obesity. Therefore, an adipose tissue-mediated atrial remodeling could also be considered in the context of MAFLD.

### 4.2 Atrial fibrillation recurrence

In our study, the impact of MAFLD on AF recurrence was assessed. We first report an incidence of AF recurrence of 42.8% at 418 days of median follow-up. Such AF recurrence incidence is consistent with previous AF ablation studies including similar proportions of persistent and paroxysmal AF: in the open-label multicenter clinical trial CABANA (63) (n = 1,108), AF recurrence was observed in 49.9% of the patient, of which 16% had persistent or long-standing persistent AF, while we found 13.6% in our study.

The impact of metabolic disorders on AF ablation outcomes has been previously demonstrated. In the LEGACY trial (64), 355 overweight patients with non-permanent AF were classified according to body weight loss (median follow-up ~48 ± 18 months). Weight loss of 10% or more accomplished an increase by a factor 6 probability of arrhythmia-free survival (adjusted OR: 6; 95% CI: 3.4 to 10.3). Accordingly, a recent study showed a similar impact of weight loss of 10% or more on AF recurrence after ablation among MAFLD patients (log-rank = 27.90; p < 0.0001) (65). This latter article by Donnellan et al. (65) also suggested that MAFLD mediates the effect of metabolic disorders on AF ablation outcomes. In this study, the authors found a higher rate of AF recurrence (56% vs 21% at 60 months) using liver imaging to diagnose steatosis presence in MAFLD. Interestingly, we did not find this effect comparing MAFLD patients to “NoMAFLD” patients using a clinico-biological score (i.e., FLI), suggesting that this effect is indeed driven by liver steatosis and not by one of the components of the score such as the BMI. Using the NFS to stratify patients according to the probability of liver fibrosis, Donnellan *et al.* reported a higher arrhythmia recurrence with a high probability of liver fibrosis (82%) as compared to patients whose risk of fibrosis is excluded (31%) (log-rank = 11.70; p = 0.003.) Our data are therefore in accordance with those from Donnellan et al. in a different population of MAFLD patients.

For the first time, we provide insights regarding the characterization of AF recurrence after AF ablation in MAFLD patients. Interestingly, we observed that MAFLD patients presented a higher AF burden when recurrence occurred as shown by the increased proportion of permanent AF following AF recurrence. This clinical observation may be secondary to the atrial remodeling observed in these patients leading to long-lasting AF episodes. Therefore, a poor metabolic profile associated with a high risk of liver fibrosis could be considered a risk marker of atrial remodeling and poor related clinical outcomes.

### 4.3 Future perspectives

The association between MAFLD and atrial remodeling raises several questions regarding the management of patients scheduled for AF ablation. On the one hand, the impact of intensive management of the metabolic syndrome prior to AF ablation is likely beneficial to reducing recurrence risk and may even justify postponing the procedure. On the other hand, a personalized AF ablation strategy in MAFLD patients with severe liver fibrosis could be considered, with a more extensive approach (e.g., additional lines and/or a combined approach with vein of Marshall ethanol infusion), especially in the setting of persistent AF. Therefore, further clinical studies assessing the impact of AF ablation strategies after MAFLD scoring are needed to address these questions.

### 4.4 Study limitations

In both cohorts, patients were classified under the MAFLD spectrum, thus including mild-to-moderate alcohol consumption and other cardio-metabolic pathologies. With the use of this classification, it is more difficult to attribute the observation specifically to the liver, but the data are certainly more relevant to clinical practice with patients suffering from metabolic syndrome (1). Liver status was determined using two non-invasive biological scores (FLI and NFS) in accordance with the first steps of NAFLD diagnosis recommendation (21), given that liver imaging (elastography or MRI) is not a common practice among cardiologists and liver biopsies are not a possibility in such cohorts, and to be consistent with a previous liver fibrosis classification (22). This classification results in an “at risk” classification of patients, without being able to affirm their real liver status. Moreover, it results in a gray area (“indeterminate fibrosis”) for the majority of patients. The BMI was not included in our Cox model since this variable was included in the FLI and NFS formula. Therefore, further studies should assess the impact of liver fibrosis on AF ablation using alternative liver fibrosis assessment tools (e.g., transient elastography) to better integrate such confounders. Moreover, we had few patients in the most severely affected group in both cohorts, resulting in a lack of statistical power. Nevertheless, due to the low number of “MAFLD w/fibrosis” patients indicated for invasive intervention, a much larger cohort would be needed. The impact of the duration of MAFLD was not integrated in the current analysis. Given the complex dynamic process involving metabolic disorders and fibrotic processes, further studies will be of interest to specifically investigate the impact of MAFLD duration on LA remodeling. Similarly, the duration of AF history was not available in our cohort, as it would require intensive cardiac monitoring. Nevertheless, the AF subtypes still significantly correlate with the overall AF burden as shown in previous studies (66). Finally, the screening of AF recurrence was not based on a predefined intensive screening strategy but on routine clinical practice. Therefore, this could result in a significant rate of non-detected AF recurrences, however counterbalanced by the relative long-term follow-up of the cohort reducing the probability of non-detected AF recurrences.

## 5 Conclusion

Patients presenting MAFLD at risk for liver fibrosis exhibit an increased LA remodeling with impaired hemodynamic, electrophysiological, and histopathological properties. These patients also exhibit a higher risk for AF recurrence following catheter ablation. The impact of the management of MAFLD on LA remodeling and AF ablation outcomes should be assessed in dedicated studies.

## Data availability statement

The raw data supporting the conclusions of this article will be made available by the authors, without undue reservation.

## Ethics statement

The studies involving human participants were reviewed and approved by Comité de Protection des Personnes/DRN. The patients/participants provided their written informed consent to participate in this study.

## Author contributions

Patient inclusion and follow-up: TD, JR, CK, FB, AV, and DK. Echography: AC, SA, and SS. Biochemical assay: PM. Study design: RD, BS, DM, SN, LB, EW, ZG, and DD. Wrote and/or revised manuscript: RD, BS, DM, and SN. All authors contributed to the article and approved the submitted version.

## Funding

This work was supported by the French National Agency programs PreciDIAB grant (ANR 18-IBHU-0001), the European Genomic Institute for Diabetes (EGID, ANR-10-LABX-0046), and PreciNASH (ANR-16-RHUS-0006). BS was a recipient of an ERC Advanced Grant (no. 694717).

## Acknowledgments

We thank the BICeL-(UMS2014-US41) cell imaging platform for the access to the equipment and the technical advice provided.

## Conflict of interest

The authors declare that the research was conducted in the absence of any commercial or financial relationships that could be construed as a potential conflict of interest.

## Publisher’s note

All claims expressed in this article are solely those of the authors and do not necessarily represent those of their affiliated organizations, or those of the publisher, the editors and the reviewers. Any product that may be evaluated in this article, or claim that may be made by its manufacturer, is not guaranteed or endorsed by the publisher.
