# Supplementary material for: High liver fibrosis scores in metabolic dysfunction-associated fatty liver disease patients are associated with adverse atrial remodeling and atrial fibrillation recurrence following catheter ablation
Source: Front Endocrinol (Lausanne). 2022 Aug 31;13:957245. doi: 10.3389/fendo.2022.957245 (PMC9471263; doi:10.3389/fendo.2022.957245)
Supplement: Supplementary file 1 [file DataSheet_1.pdf]

Supplementals

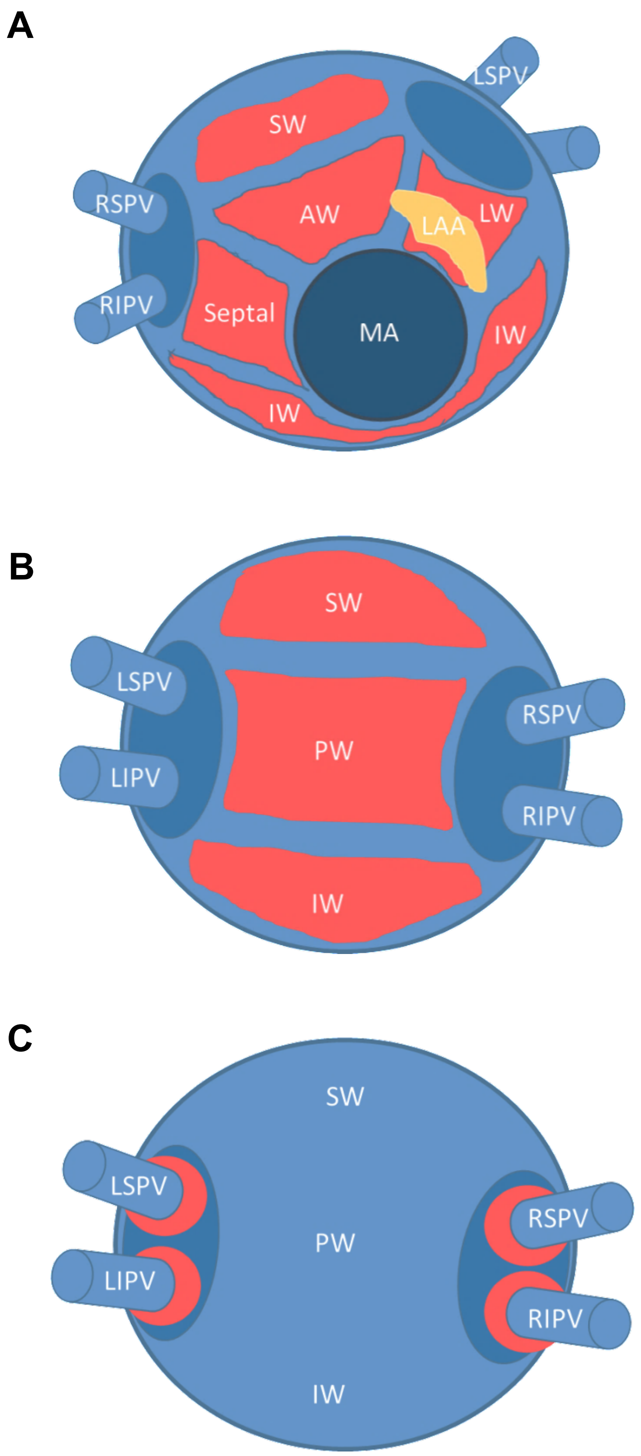

Figure S1: Assessment of LA bipolar voltage maps. LA segmentation. (A: Left Anterior Oblique 50° view; B – C: Posterior-Anterior view). MA, mitral annulus; AW, Anterior wall; SW, Superior wall; IW, Inferior wall; LW, Lateral wall; PW, Posterior wall; LSPV, left superior pulmonary vein; LIPV, left inferior pulmonary vein; RSPV, right superior pulmonary vein; RIPV, right inferior pulmonary vein.

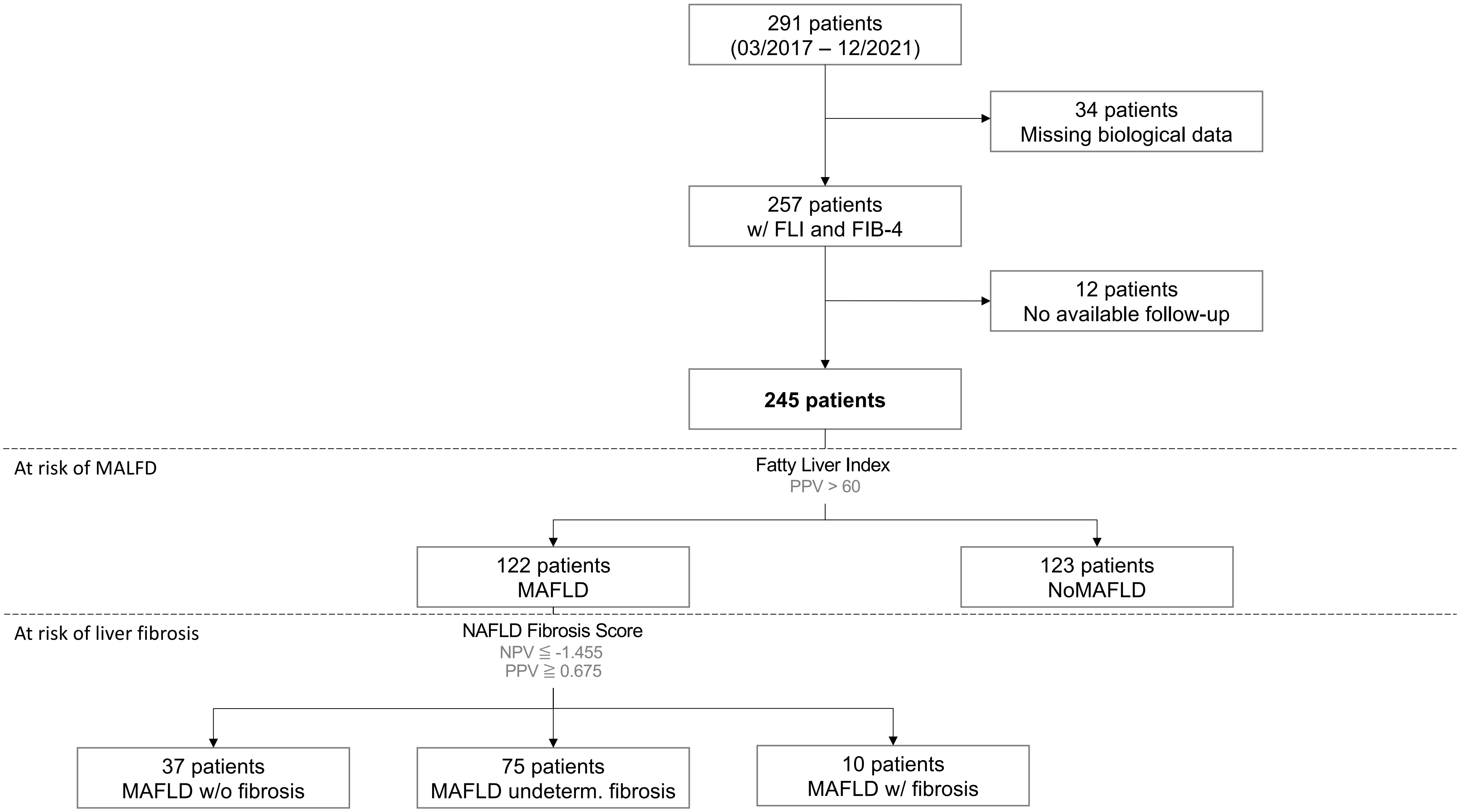
291 patients
(03/2017 – 12/2021)
34 patients
Missing biological data
257 patients
w/ FLI and FIB-4
12 patients
No available follow-up
245 patients
At risk of MALFD
Fatty Liver Index
PPV > 60
122 patients
MAFLD
123 patients
NoMAFLD
At risk of liver fibrosis
NAFLD Fibrosis Score
NPV ≦ -1.455
PPV ≧ 0.675
37 patients
MAFLD w/o fibrosis
75 patients
MAFLD undeterm. fibrosis
10 patients
MAFLD w/ fibrosis

Table S1: Baseline characteristics of patients referred for heart surgery.

	NOMAFLD	MAFLD W/O FIBROSIS	MAFLD W/ SEVERE FIBROSIS
n	12	5	3
Criteria	FLI<60	FLI>60 ; NFS<-1.455	FLI>60 ; NFS>0.675
Age (years)	66.5 [47.3-71.5]	64.0 [54.0-73.0]	62.0 [59.0-75.0]
BMI	26.4 [23.0-28.9]	29.6 [28.2-33.1]	32.8 [31.3-54.9]
HOMA-IR	0.58 [0.46-1.19]	1.03 [0.83-1.60]	0.95 [0.43 - 2.46]
SBP (mmHg)	137 [125-149]	126 [121-139]	125 [120-141]
DBP (mmHg)	84 [78-92]	74 [73-84]	73 [63-92]
LVEF (%)	67 [60-73]	66 [59-69]	63 [60-67]
LAA (cm^2)	24 [22.5-29.5]	32 [27-33.5]	41 [24-45]
MVGi	51 [42-58]	55 [50-63]	59 [51-69]
AoS, n	9	4	2
Mitral, n	3	1	1

Abbreviations: BMI=Body mass index ; SBP=Systolic blood pressure ; DBP=Diastolic blood pressure; LVEF=Left ventricular ejection fraction ; LAA=Left atrial area ; AoS=Aortic stenosis.

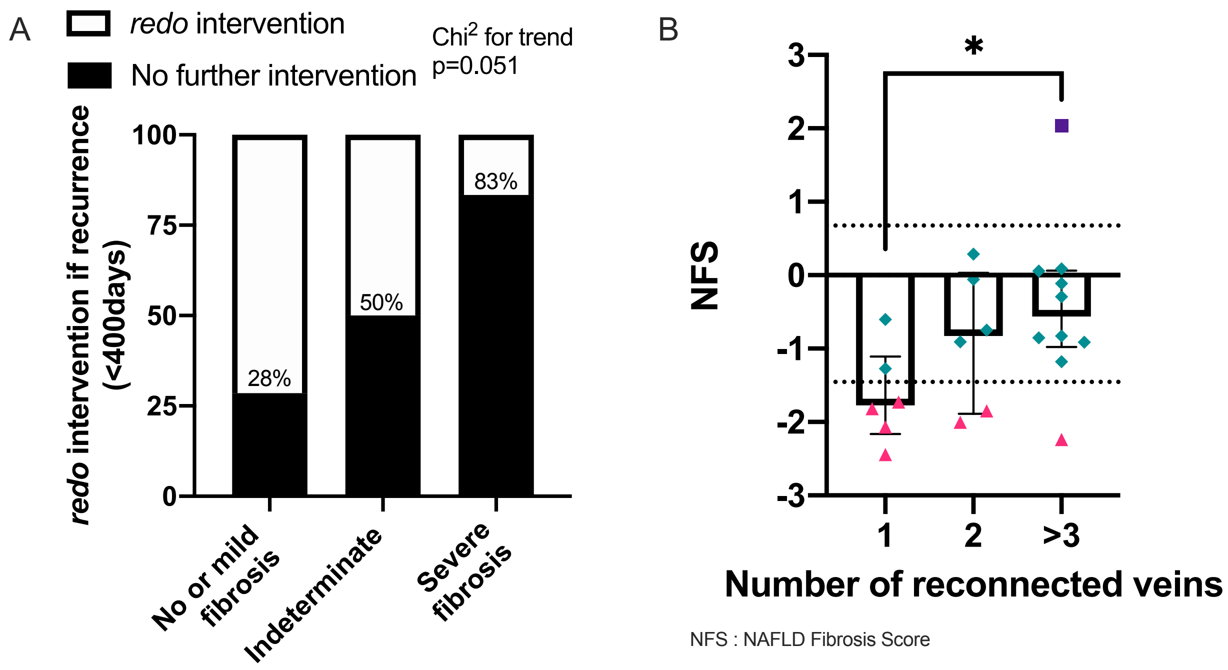

NFS : NAFLD Fibrosis Score
